# Making it normal for ‘new’ enrollments: effect of institutional and pandemic influence on selecting engineering institutions under the COVID-19 pandemic situation

**DOI:** 10.1016/j.heliyon.2021.e08217

**Published:** 2021-10-19

**Authors:** Prashant Mahajan, Vaishali Patil

**Affiliations:** a R. C. Patel Institute of Technology, Shirpur, India; b RCPET's, Institute of Management Research and Development, Shirpur, India

**Keywords:** Engineering education, Choice characteristics, Institutional influence, Pandemic influence, Suitability under the COVID-19

## Abstract

The COVID-19 pandemic has forced Indian engineering institutions (EIs) to bring their previous half-shut shades completely down. Attracting new admissions to EI campuses during the pandemic have become a ‘now or never’ situation for EIs. During crisis situations, EIs have struggled to return to their normal track. The pandemic has drastically changed students' behavior and family preferences due to mental stress and the emotional life associated with it. Consequently, it has become the need of hour to examine the choice characteristics influencing the selection of EIs during the COVID-19 pandemic.

The purpose of this study is to critically examine institutional influence and pandemic influence that affects students’ choice about engineering institutions (EIs) during COVID-19 pandemic situation and consequently to study relationships between them. A quantitative research, conducted through a self-report survey composed by a closed-ended structured questionnaire was performed on the students who were recently enrolled in the EIs (academic year 2020–2021) belonging to North Maharashtra region of India during the pandemic.

The findings of this study have revealed that institutional and pandemic influence have directed EI choice under the COVID-19 pandemic. It is also found that pandemic influence is positively affected by institutional influence. The study demonstrated that EIs can attract new enrollments by repositioning their institutional characteristics that regulate pandemic influence. The study can be a measuring tool for policy makers to attract new enrollments under pandemic situation.

## 1 Introduction

Worldwide, engineering education is viewed as a career of progressive growth that has the potential to shape human skills (Blom and Saeki, 2011), social and quality of life (Rojewski, 2002), economy of the country (Cebr, 2016) and overall development of the country (Downey and Lucena, 2005). Thus, engineering education has proven to be a key factor for the sustainable and profitable development of society. It encourages global competitiveness through engineering inventions for the benefit of society at large. Although the demand for engineers remains relatively high throughout the world, there are few aspirants willing to pursue engineering education. Creating an upswing for interest and fondness that makes students inclusive of engineering studies has been a challenge over the past decades. Reports on engineering education about declining enrollments in the context of India (AICTE New Delhi, 2021) and diminishing interest and trends worldwide (UNESCO, 2010) have signaled a warning for the overall development of hi-tech society. In India, the gap between available seats (capacity) at the entry level and actual admissions in degree engineering is widening year by year, leaving approximately 5.9 lakhs seats vacant in 2019–2020. All India Council for Technical Education, New Delhi, an apex body for governing technical education, indicated that approximately 45% of seats remained vacant in the 2019–2020 academic year, which was earlier noticed to be 38% in 2012–2013. Most of the studies have verified that this situation is due to problems pertaining to awareness, attraction, recognition of needs and service offers (Kamokoty et al., 2015; Upadhayay and Vrat, 2017).

Selecting an institution, as acknowledged by previous literature, is a subtle and complex phenomenon (Hossler et al., 1989a) that involves a multifaceted and inconsistent set of institutional influencing characteristics (Chapman, 1981; Obermeit, 2012). It implicates a challenging progression for institutions as well as aspirant students (Hemsley-Brown and Oplatka, 2015b) and requires greater efficiency and effectiveness to make a concluding decision. Decisions regarding ‘institutional choice’ can change students' lives forever (Iloh, 2019) as well as lifelong performance of the institutions. Selecting an engineering institution (EI) has not received much consideration but is practically missing in the literature, as the research drift appears to be inclined towards general higher education addressing psychology, sociology, and economics disciplines (Paulsen, 1990). Today, most EIs in India with lower enrollments are in vilest positions due to the absence of practicing students' assessment in regard to their choices and needs. Engineering education is highly contrasted with respect to the multidimensional thoughts of students and institutional offers related to the quality of staff and teaching-learning, infrastructure and facilities, course value and delivery, and outcome benefits.

### 1.1 Statement of the problem

There is certain evidence that higher education needs to be drastically reformed due to unforeseen situations or crises due to political and economic changes arising due to natural disasters (Schuh and Santos Laanan, 2006) and pandemics (Kim and Niederdeppe, 2013). In such situations, institutions have struggled to return on their normal track. Aristovnik et al. (2020) revealed that the COVID-19 pandemic has changed students’ emotional and personal lives and has also changed their preferences and habits in regard to the selection of higher education. The survey conducted by The International Association of Universities discovered that COVID-19 will affect future enrollment for upcoming academic years (IAU, 2020). Consequently, it becomes the need of hour to examine the choice characteristics influencing the selection of EIs during the COVID-19 pandemic situation. It also holds great practical importance for policy makers of EIs to decide strategies to attract new enrollments during pandemic situation.

### 1.2 Objective of study

As informed by the evidences and problems discussed above, the main objective of this study is to critically examine choice characteristics related to institutions and pandemics that influence students’ choice for EIs during the pandemic and consequently to explore relationships between institutional and pandemic influence arising due to COVID-19 situation. The above objective is underpinned by the following research questions referring to the selection of an EI during the COVID-19 pandemic.

1. What are the important characteristics associated with institutional and pandemic influence that affects prospective students' decisions of selecting EIs?
2. How are institutional influence and pandemic influence coupled?

## 2 Literature review

This study embraces a systematic review (Bearman et al., 2012) that progressed gradually through extensive searching, selecting and integrating literature that has explored the evolution and influence of choice characteristics responsible for the selection of an institution. The literature review revealed that the institute choice process has reformed over time in accordance with ecological changes (Jackson, 1988) and informed way of awareness and understanding of institutional facilities (Nora and Cabrera, 1992). To make a pathway for prospective students, institutions should understand who students are and what they expect from them and how their expectations can be met by educational offers (Han, 2014). Hemsley-Brown and Oplatka, 2015a, Hemsley-Brown and Oplatka, 2015b, Hemsley-Brown and Oplatka, 2015c learned that despite ample literature, there is no assured list of choice characteristics that influence choice decisions and confirm students picking up a specific institute. The following section describes at length the characteristics linked to institutional and pandemic influence that are accountable for students’ choice decisions.

### 2.1 Institutional influence

Institutional influence is a set of characteristics that magnetizes prospective students towards institutions for their higher studies. These characteristics are clustered on financial vs nonfinancial offers, academic vs nonacademic facilities and services, and tangible vs intangible factors (Hossler et al., 1989b) (Yamamoto, 2006), which are reviewed below.

#### 2.1.1 Proximity to hometown

Proximity relates to the nearness of hometown from the institution. Being close to an institution is a significant factor for students in selecting an institution (Turley, 2009). It also increases the chance of acceptance for the particular institution (López Turley, 2009), as distance travel is associated with cost, time and efforts (Chapman, 1981). In the case of engineering study, due to a heavy workload, proximity can provide students extended hours for their study at home, and enough time for social and other activities.

#### 2.1.2 Location and locality

Location and locality characterizes ambient conditions, speciousness and functional accessibility (Bitner, 1992) and are swaying characteristics in making institutional choices (Gibbs and Knapp, 2012). Location gives the impression of institute's site and its connectivity from hometown, while locality refers to culture, amenities, and facilities available in surrounding place wherein the institution is located (Mahajan and Golahit, 2019). Overall, it is credited with suitability, vicinity, attractiveness, accessibility, cost-effectiveness, safety and security (Hannagan, 1992; Kotler and Fox, 1995).

#### 2.1.3 Image and reputation

Image and reputation in public minds play a significant role in differentiating institutions (Imenda et al., 2004) and is measured as one of the utmost characteristics in influencing institution choice (Briggs, 2006; Wadhwa, 2016). It is composed of a spectrum of small reputes, such as academic and nonacademic characteristics belonging to institutions (Lafuente-Ruiz-de-Sabando et al., 2018). In the review of the literature conducted by Hemsley-Brown and Oplatka (2015c) and in most of the research such as Maringe (2006b), the image and reputation provides first sight impression and creates positive feelings in decision makers’ minds, even if nobody is confronted with the institutions.

#### 2.1.4 Faculty profile

Faculty profiles in terms of their qualifications, skills, competency and experience (Imenda et al., 2004) exert a significant influence on the students (Mazzarol and Soutar, 2002; Soutar and Turner, 2002). Faculty ought to be profiled with high-quality teaching (Woolnough, 1994) and should be a well designer (De Courcy, 1987). Similarly, they should be well-inspired, well informed, passionate, open minded, and responsive (Voss et al., 2007) to transform knowledge and to assist students in real-world exposure (Bhattacharya, 2004). Magnell et al. (2017) mentioned the importance of faculty attitudes in assisting students in availing engineering pathway.

#### 2.1.5 Alumni image

Alumni are the tangible outcome of the institutions, and hence, alumni concerns are important criteria in measuring the performance of EIs. Alumni achievements are often exploited to exemplify the importance, eminence and image of institutions (Saunders-Smits and de Graaff, 2012) and is considered as a key criteria for selecting an institution (Ho and Hung, 2008). Historically, alumni images with economic, market and social standing at all times have added glory to the reputation of their institutions and hence have become benchmarking standards for prospective students (Pucciarelli and Kaplan, 2016).

#### 2.1.6 Campus placements

Employment prospects are the potential outcomes and benefits that prospective students and their families seek against the time, effort and money invested in the institutions (Hemsley-Brown and Oplatka, 2015c; Maringe, 2006a). The transition from education to employment is the straightforward motive of every student opting engineering study (Baytiyeh and Naja, 2012) and has been tested to be one of the most influential characteristics in making institutional choices (Malgwi et al., 2005). Most premium EIs uphold alliances between industry and academia through the series of employment activities that deal with campus placements. Employment activities play a major role in boosting employability skills (Markes, 2006) and accelerating industry-academia connections (Baytiyeh and Naja, 2012) to create opportunities for campus placements.

#### 2.1.7 Quality education

Quality of education is a prime, discriminating, and prominent influencing characteristic designed to stay ahead in a competitive market and to make a place in the minds of stakeholders. Several studies (Pandi et al., 2014; Sakthivel and Raju, 2006a; Sayeda et al., 2010) have emphasized the importance of quality education in regard to the holistic development of institutions and in making choice decisions for students (Kallio, 1995; Mourad, 2011). Several aspects, such as academic standards, industry linkages, and campus placements contribute to the quality of education (Mahajan et al., 2014). Furthermore, for some researchers, it is enhanced by providing better course delivery (Trum, 1992), infrastructure facilities (Sayeda et al., 2010), faculty (Gambhir et al., 2013), quality services (Viswanadhan, 2009), and academic and nonacademic concerns (Jain et al., 2013; Owlia and Aspinwall, 1998). Overall, it has a two-fold effect in terms of tangible and intangible outcomes (Natarajan, 2009).

#### 2.1.8 Infrastructure and facilities

Numerous studies like Nyaribo et al. (2012), Sahu et al. (2013) and Price et al. (2003) have mentioned importance of infrastructure and facilities in engineering education. It consists of buildings, equipment, infrastructure and amenities that are tangible possessions reflecting the capacity of institutions that streamline the performance of curriculum delivery (Palmer, 2003). It can provide love-at-first-sight and becomes on-the-spot physical evidence for prospective students (Philip Kotler et al., 2002; Mahajan and Golahit, 2019). Delivering a curriculum without the physical existence of infrastructural assets and facilities is not possible for EIs, as curriculum delivery in regard to engineering education is more technical in nature.

#### 2.1.9 Safety and security

Safety on the campus is the provision made in regard to residential, physical health, and life concerns to ensure the wellbeing of students (Ai et al., 2018), whereas security, as a broad term, covers human rights, emotions and cultural values associated with students (Calitz et al., 2020). Studies such as Elliott and Healy (2001) and Peters (2018) have exposed that students contemplate safety and security based on wellbeing and humanized culture, whereas, Calitz et al. (2020) revealed that it is associated with decisions about the selection of institutions. The students feel comfortable with the health services, emergency and situational provisions delivered by the institutions (Sakthivel and Raju, 2006b).

#### 2.1.10 Curriculum delivery

In engineering education, curriculum delivery is the most influential characteristic and is found to be the first priority in selecting an EI in most studies, such as Moogan and Baron (2003). It is associated with execution of a planned pedagogy supported by intangible services and tangible facilities that ensures continuous transfer of knowledge (Case et al., 2016). It can add glory to the institutions, if delivered as per the needs of students but can be unpleasant for the students if not delivered properly. Curriculum delivery involves multi-modal approaches such as online (Alawamleh et al., 2020), hybrid or blended (Sia and Adamu, 2020; Tan, 2020) and regular onsite delivery depending on the situational crises. Although all have their own advantages and disadvantages in regard to the involvement of theory vs practical, technology vs human, and competency skills achieved. However, the degree to which it facilitates accessing, practicing and implementing knowledge is more important (Shay, 2014). To attract enrollments, delivery of engineering curriculum is to be considered a backbone of EIs that transforms engineering knowledge into practical applications (Hemmo and Love, 2008).

#### 2.1.11 Value for money

Value for money is an intangible characteristic and deemed to be an anxiety for students that influences their decision of selection of institutions. In engineering studies, the nature of cost is differential and includes tuition, travel, residential and food cost, and day-to-day academic cost, which are more expensive than other higher education disciplines. Some studies have exhibited the cost of education as a package of rewarding value benefit entailing, value and quality (Ivy, 2008; Joseph et al., 2005), time and effort (Kotler and Fox, 1985), and effort and opportunity (Wu et al., 2020). The degree of engineering, employment opportunities, skills gained, and social status are the foreseen values for the students against their financial investment.

### 2.2 Pandemic influence

Pandemic influence referring to this study is all about COVID-19 pandemic situation triggered due to corona virus. It is an external influence that affects customers' behavior due to psychological perceptions about the situation (Belk, 1975). COVID-19 disease was first discovered in December 2019 which causes respiratory illness spread though small saliva in the form of droplets and aerosols occurring due to close human contacts (Ciotti et al., 2020). As indicated by the World Health Organization, physical and social distancing is the only credible way to constrain its spread. It has taken out higher education by storm and hence turns out to be the most challenging condition in the history of engineering education. A US-based study (Aucejo et al., 2020) showed that the influence of the COVID-19 pandemic on higher education is extremely heterogeneous. In the past, during situational crises, Rosenthal et al. (2014) emphasized on appropriate curriculum delivery, and Kim and Niederdeppe (2013) suggested students’ support systems as the important aspects in normalizing the situation and continuing pedagogy.

In India, unlocking pandemic restrictions was started in August 2020. The admission process for admitting new students in EIs for the academic year 2020–2021 in the state of Maharashtra, India was completed in January 2021. EIs were able to commence academic sessions for newly joined students from February 2021, as per the guidelines of authorities (UGC, 2021) and the norms of State Government, that restricted onsite pedagogy with a 50% batch size on a rotation basis. Meanwhile, there were many pros and cons all over the world, about curriculum delivery during the COVID-19 pandemic situation. To some authors, online delivery is most suitable during the pandemic to continue education (Gautam and Gautam, 2020; Liguori and Winkler, 2020). However, it has been adversely condemned for various reasons, such as technology availability, academic loss and ongoing interest (Bird et al., 2020; Tesar, 2020; Zia, 2020). Some authors have suggested hybrid/blended delivery (Rashid and Yadav, 2020; Sia and Adamu, 2020) as a solution to continuing pedagogy during the pandemic. Aristovnik et al. (2020) revealed that the pandemic with emotional life has also affected students’ behavioral characteristics in terms of their likings and preferences. Thus, pandemic situation has stressed prospective students to think more about better accessibility and suitability. Therefore, there is an urgent need for policy reforms that sustain the mental health and social emotions of students (World Health Organization, 2020). UNESCO (2020) judged that education has to be redefined or reduced, replaced or enhanced to engage students, particularly to avoid academic, social and emotional loss. For that reason, Chadha et al. (2020), in a recent study on UK engineering students, articulated that there is more need to implement new reforms to ensure that engineering education and the students should not go down its normal pathway.

Thus, the pandemic influence referring to this study is EIs' efforts and provisions for making engineering education sustainable and justifiable by providing suitable facilities and support services that mitigate the impact of the pandemic on students’ pedagogy by following government guidelines about social distancing.

### 2.3 Research gap and significance of study

Many researchers have notarized a variety of characteristics influencing institutional choice decisions, originating due to different cultures, economic and social reforms, but all were administered under nonpandemic situations. Many researchers felt that students’ behavior changed during the COVID-19 pandemic, and there is urgency to reposition the framework of policies, which demand future research that urges exploring institutional choice characteristics and pandemic influence during the pandemic.

Moreover, there is no such research to date that provides knowledgeable relationships between students' perceptions of EI selection during the COVID-19 pandemic. The importance and timeliness of this study is boundless, as it is aimed to explore radical changes that materialized in students’ choice characteristics during the COVID-19 situation.

### 2.4 Conceptual framework and hypothetical model

The literature review has shown that choice decisions are based on attractive and beneficial offers made by institutions in regard to tangible facilities and intangible services. However, during the COVID-19 pandemic, the process of evaluating EIs involved a more intellectual and meticulous screening of institutional characteristics. In pandemic situation only these characteristics can overcome external influence (pandemic influence), by providing greater suitable and accessible educational services that constrains the spread of corona virus.

**Figure 1:**
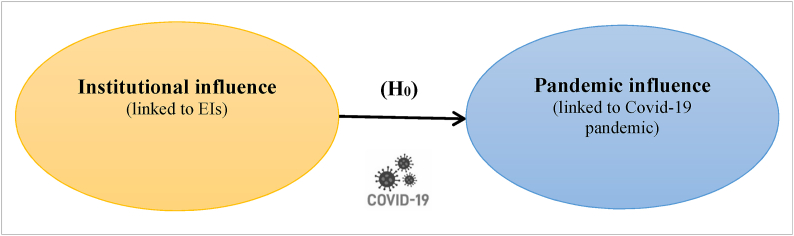
Hypothetical path model of choice influencing characteristics under the COVID-19 pandemic. Source: Own.

Based on the theoretical and conceptual framework as stated above and the specified objective of the study, the following hypothetical model (refer to Figure 1) will stand for answering the research questions. Following null Hypothesis will be validated based on students’ perceptions.

Hypothesis (H_0_) There is no significant relationship between students’ perceptions on institutional influence and pandemic influence when selecting EIs under the COVID-19 pandemic situation.

## 3 Research methodology

### 3.1 Research design

This study is about an educational dilemma associated with EI choice, particularly during the COVID-19 pandemic. A literature review aligned with the objective of this study has enabled this study to implement quantitative methods due to their ability to frame hypotheses (Borrego et al., 2009), capabilities to operate on multivariate statistical data (Creswell and Creswell, 2017), ability to analyze relationships with definiteness (Creswell, 2012b), reliability (Steckler et al., 1992) and success in educational research (Tight, 2012).

The judgment of what students truly receive from the institutional service against their expectations is often based on the evaluation of students’ perceived experience (Yelkur, 2000). Therefore, this study has considered students as the primary customers of higher education (Maringe and Gibbs, 1989) and is set to report their perceived experience about their pathway to engineering institutions during the pandemic. Therefore, students who recently enrolled in EIs during the COVID-19 pandemic situation were selected as a population of this study. Primary data are collected using a survey method that is most suitable for collecting preferences and choices from a large number of responses (Kotler et al., 2016).

The admission process for the first-year degree engineering program for 2020–2021 was conducted under the control of competent authority of Maharashtra State, India, and came to end in January, 2021. Thirty nine EIs offering degree program in engineering and technology situated in the North Maharashtra region of India were chosen as the sampling frame of this study. Newly joined students (academic batch 2020–2021) admitted in these EIs have been chosen purposefully, as a sampling units of this study as they recently have experienced decision making process of selecting their EIs under the COVID-19 pandemic period. Purposive sampling has been chosen decisively because of the knowledge and judgment of researchers (Creswell and Creswell, 2017), to report special situations (Neuman, 2013) like pandemic and to investigate of new issues (Etikan et al., 2016) about ‘EI choice’ during the COVID-19 pandemic. Total of 4300 e-mail addresses of admitted students representing population of this study from selected sampling frames (EIs) were collected online during February 2021. Permission from each of EIs to utilize students' data to administer self-report survey was also obtained online. Before starting this survey, ethical approval was obtained from an Institutional Ethical Committee, R. C. Patel Institute of Pharmaceutical Education and Research, Shirpur (India) that confirmed that informed consent was obtained from all human participants involved in this study and this research study meets the national guidelines and protocols for research on human objects. To make students more responsive, a self-report survey (Kolb, 2008) was conducted over the internet via the Google Form tool representing a questionnaire of this study during February, 2021. By this time, students were well known about the pandemic situation and have experienced the social distancing during pandemic situation.

During the pandemic, a self-report survey was very useful, as it avoided direct contacts with the respondents during the pandemic but at the same time ensured its reach to the expected respondents (students). This method also assisted in receiving responses quickly by providing respondents with better flexibility in time and place and avoided researcher bias. Out of 4300 admitted students (population of area under study), the survey received online responses with the consent to publish the responses and research findings from 922 students at a response rate of 21% at the end of February 2021. All gathered responses which were found complete and valid and hence accepted for further analysis. Among the respondents, there were 596 male (65%) and 326 female (35%) students by gender. Based on geographic location, 609 students (66%) belonged to rural and 313 students (34%) belonged to urban locations. There were 685 students (74%) representing higher social class and 237 students (26%) were from lower social class based on their socio-economic status. All respondents were aged in between 18 to 20 years and had qualified their Grade XII examination in Science stream (Higher Secondary School examination). Creswell (2012a) has recommended a sample size of at least 20 samples per variable. A sample size of 922 for assessing twelve variables associated with this study, which derives 77:1 (samples per variable), is sufficiently defensible against the traditional arbitrary ratio of 20:1 (Maxwell Scott, 2000).

### 3.2 Scale design and data collection

A quantitative survey is administered with a list of structured closed-ended questionnaires prepared as per the guidelines provided by Navarro Sada and Maldonado (2007) and Ary et al. (2010). The questionnaire was initiated with an introductory part, Section 1, explaining the purpose and importance of the study. After that, approval regarding utilizing and publicizing survey responses publicly, was requested. The next sections were initiated only if the respondents had granted the approval. Section 2 presents questions on students' personal characteristics, such as gender, age, social class, and native place. Section 3 was associated with choice influencing characteristics, which were evidenced under a literature review and recommended by academic experts. This section encompassed twelve items symbolizing institutional characteristics that influence students’ decisions about the selection of their EIs. In this regard, students are asked to rate these questions on 5-point Likert scale to capture a range of the intensity of their perceived experience for the items; proximity to hometown, location and locality, image and reputation, faculty profile, alumni profile, campus placements, quality education, infrastructure and facilities, safety and security, curriculum delivery, value for money and suitability under the COVID-19 pandemic situation. Before entering the actual survey, the validity and reliability of the questionnaire were tested through pilot testing (Kenneth, 2005) on few samples selected from sampling units, to understand its language and sequence of questions. After pilot testing, questionnaire then utilized for conducting actual survey.

## 4 Data analysis and statistical results

Making EI choice is a new encounter and difficult for prospective students under the COVID-19 pandemic situation. In such a situation where choice influencing characteristics are unknown and their relationships are unfamiliar, the data analysis is executed by a two-step approach (Anderson and Gerbing, 1988). To determine the relationship between institutional influence and pandemic influence, exploratory factor analysis (EFA) and structural equation modeling (SEM) were performed. In the first step, factor analysis by EFA is performed to develop constructs (latent variables) from item scales (observed variables), followed by confirmatory factor analysis (CFA) performing structural equation modelling (SEM) in second step to predict the relationships between the extracted constructs (Byrne, 2013). Both EFA and CFA were performed on the entire sample to confirm CFA and to achieve better fit for SEM as per hypothesized relationships. The data were analyzed and analyzed with the techniques available in the statistical software SPSS 25.0 and AMOS 25.0. Before arriving at the EFA and SEM results, the statistical fitness of the data in terms of sample adequacy, reliability and validity were justified as discussed below.

### 4.1 Statistical fitness of data

Reliability based on internal consistency was successfully validated based on Cronbach's alpha, item-total correlation, and the split-half technique available in SPSS under reliability analysis (refer Table 1). Values of Cronbach's alpha are above 0.6 for all scale items that have confirmed scales' internal consistency (Churchill Jr, 1979) and are best fit for the purpose (Nunnally and Bernstein, 1967). Next, corrected item-total correlations, which are noticed above 0.33, indicated good internal consistency of scales (Briggs and Cheek, 1986) and are found below 0.85, which proves no potential issues on multi-collinearity (Kline, 2005). The split-half method has successfully correlated half of the scale items with the other remaining half. For both parts, the value of the Spearman-Brown coefficient has displayed the same value (0.93) within the parts, which expressed that the observed variables have more internal consistency with their latent variables (Ho, 2006). Composite reliability (CR) and average variance extracted (AVE) for each extracted latent variable derived from EFA are calculated (Refer Table 1). The obtained values are well above the acceptable level of 0.7 (Fornell and Larcker, 1981) for CR and above 0.5 for AVE (Joseph et al., 1998). Last, Tukey's test was effective in detecting no additivity, which confirmed a sufficient estimate of power.

**Table 1:** EFA results with reliability and validity.

Observed variables		Mean	Corrected Item-Total correlation	Latent variables	
Choice characteristics	Code			Component 1	Component 2
Location and locality	C1	3.992	0.745	0.761	--
Image and reputation	C2	4.044	0.759	0.799	--
Faculty profile	C3	3.964	0.805	0.847	--
Alumni profile	C4	3.906	0.789	0.822	--
Campus placements	C5	3.979	0.769	0.825	--
Quality education	C6	3.937	0.785	0.809	--
Infrastructure and facility	C7	3.911	0.794	0.805	--
Safety and security	C8	3.972	0.783	0.798	--
Curriculum delivery	C9	3.929	0.789	0.794	--
Value for money	C10	3.764	0.677	0.682	--
Proximity	C11	3.329	0.464	--	0.923
Suitability under Covid-19	C12	3.502	0.464	--	0.619
No. of scale items				10	2
Eigen value				7.105	1.052
% variance				54.369	13.603
α based on standardized items				0.944	0.627
Composite reliability				0.945	0.757
AVE				0.633	0.618
Component labeling				Institutional influence (II)	Pandemic influence (PI)

Notes: Extraction method: principal component analysis (PCA). α: Reliability Coefficient.

Rotation Method: Varimax with Kaiser Normalization.

Rotation converged in three iterations with extraction of two components.

The scale items under this study signifying influencing choice characteristics about EIs are collected from rigorous analysis of the literatures. In addition, academic experts associated with engineering education have confirmed that these characteristics are responsible for the inclusion of students in EIs. Factor loadings for all observed variables are well above 0.4, indicating that all twelve scale items are loaded strongly and significantly, confirming strong construct validity for their respective latent variables (refer Table 1). Finally, that are no scale items that have factor loadings above 0.4 across another construct (excluding own construct), which suggested that all scale items clarify sound discriminant validity (Hair et al., 1998; Ho, 2014). Because each scale item has loaded on only one latent variable, there is evidence of convergent and discriminant validity.

### 4.2 Step I - scale reduction and component extraction by EFA

EFA is performed to determine how and to what extent the observed variables are connected to their underlying component (latent variable) (Byrne, 2005). To start with EFA, all twelve choice characteristics (scale items) have been processed with varimax rotation keeping the eigenvalue above 1.0 (Ho, 2006). Overall, all scale items have demonstrated a high level of potential for being factorized, with a Kaiser-Meyer-Olkin (KMO) measure of sampling adequacy at value 0.958 which is greater than required value (>0.5) as suggested by Joseph et al. (2006). Moreover, the chi-square value of χ^2^ = 7328.117 (*df* = 66, *p* < 0.000) has shown creditable adequacy for factor analysis with Bartlett's test of sphericity (Cerny et al., 1977).

**Figure 2:**
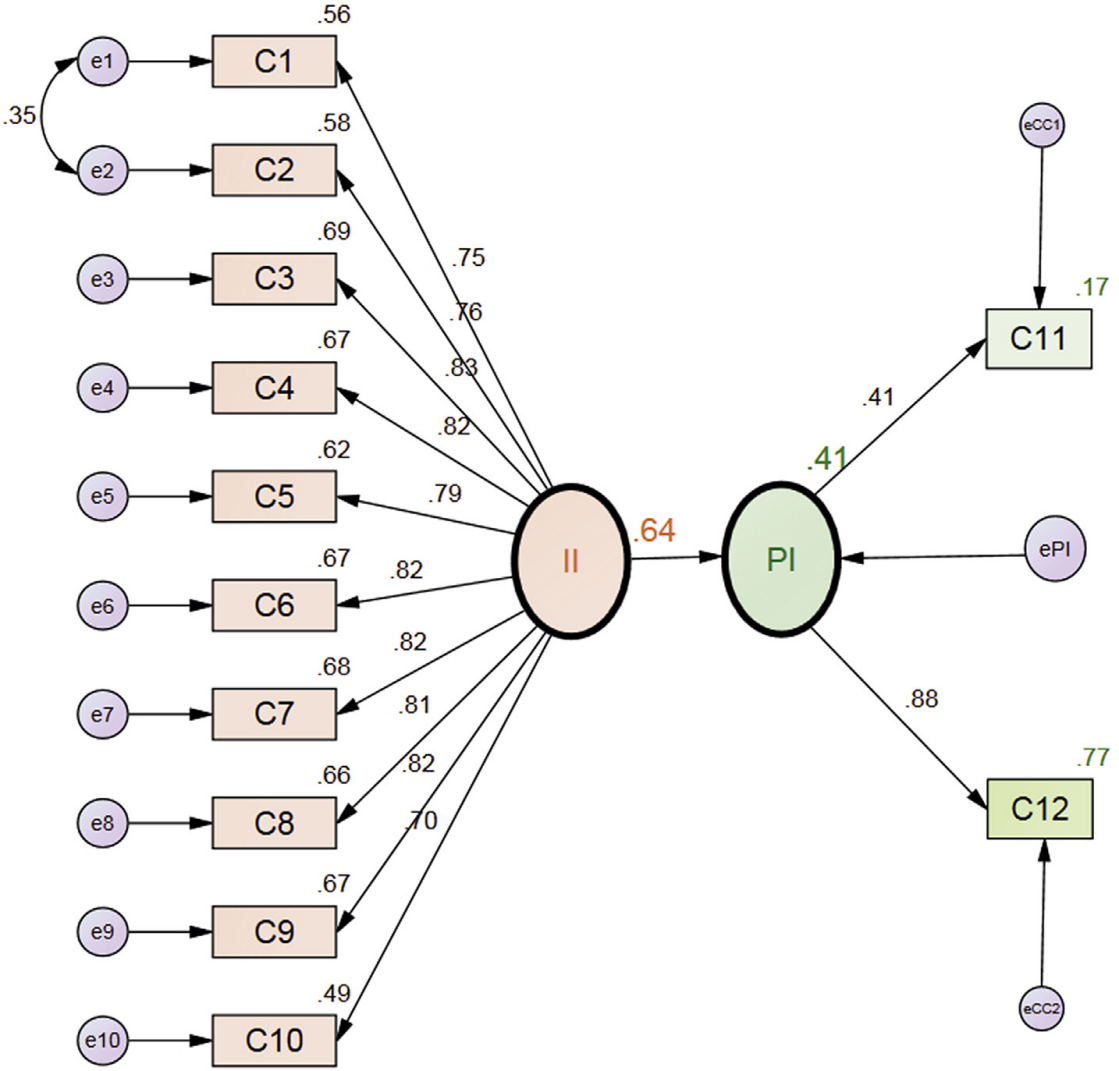
Structural equation modelling on ‘choice influencing characteristics’ during pandemic. Source: SPSS AMOS.

EFA extracted two main components which contained items with common features within components, however, noticed to be dissimilar across the components (refer to Table 1). The first component is extracted from ten scale items (C1 to C10) accounting for 59.2 percent of the variance. It is labeled ‘institutional influence’ (II), as all ten scale items represent traditional institutional characteristics that are usually accessed by students during nonpandemic situations for selecting EIs. Cronbach alpha (α) for this component is 0.944. The second component explained 8.77 percent of the variance and exhibited an eigenvalue of 1.052 (above 1.0). It is comprised of two scale items (C11 and C12) symbolizing choice characteristics associated with pandemic situation. This component is classified as ‘pandemic influence’ (PI). Cronbach alpha for this component was 0.627, which is lower than that of the previous component due to the few item scales associated with it, however, was within acceptable limits (Tavakol and Dennick, 2011). Labeling of components is created on the type of scale items it houses and its relevance to the reviewed literature on institutional choice. The factor loading for the first extracted component ranged from 0.682 to 0.847, and for the second component, it ranged from 0.619 to 0.923, showing strong construct validity. The hypothetical path model is estimated to assess the explanatory power of all independent observed variables associated with the latent variables. Then, Step II is proceeded to justify the strength and significance of the relationships by performing CFA and SEM, as discussed below (Refer Tables 2 and 3 and Figure 2).

**Table 2:** CFA estimates.

Choice characteristics (endogenous variables)	*R*^2^	Total effects based on SRW (β)	
		On account of II	On account of PI
Observed Variables			
C1	0.562	0.750	0.000
C2	0.581	0.762	0.000
C3	0.689	0.830	0.000
C4	0.667	0.817	0.000
C5	0.623	0.789	0.000
C6	0.671	0.819	0.000
C7	0.680	0.824	0.000
C8	0.660	0.812	0.000
C9	0.667	0.816	0.000
C10	0.494	0.703	0.000
C11	0.171	0.263∗	0.413
C12	0.773	0.560∗	0.879
Latent Variable			
PI	0.406	0.637	0.000

Notes: SRW, standardized regression weights; *R*^2^, squared multiple correlations; ∗, indirect effects.

Source: SPSS AMOS.

**Table 3:** CFA – Variance and relationships with internal consistency.

Variance and relationships	*B*	SE	CR	*p*-value
**Component II**				
(Variance)	0.417	0.035	12.039	<0.001
(Relationships)				
C1←II	0.988	0.045	21.873	<0.001
C2← II	0.967	0.044	22.231	<0.001
C3←II	1.058	0.044	24.171	<0.001
C4← II	1.062	0.045	23.789	<0.001
C5←II	1.003	0.044	23.014	<0.001
C6←II	1.075	0.045	23.861	<0.001
C7←II	1.063	0.044	24.010	<0.001
C8←II	1.019	0.043	23.672	<0.001
C9←II	1.037	0.044	23.785	<0.001
C10←II	1.000	--	--	--
**Component PI** (Relationships)				
C11←PI	0.562	0.068	8.282	<0.001
C12←PI	1.000	--	--	--
**Hypothesis**				
**PI←II**	**0.942**	**0.057**	**16.434**	**<0.001**

Notes: Relationship: observed variable and latent variable, *B*, regression weights; SE, standard error; CR, critical ratio.

Source: SPSS AMOS.

### 4.3 Step II – executing the measurement model through CFA and SEM

CFA and SEM are performed according to the guidelines suggested by (Byrne, 2013; Schumacker et al., 2010). The CFA method is employed to examine the factor structure of all influencing characteristics (observed variables), whereas SEM is used to model a network of structural relationships that exist between observed variables and latent variables.

In the beginning, the model is specified as per the results of EFA and hypothetical path model. Pathways were drawn accordingly. To prove the Hypothesis, a one-way directional path is connected from II towards PI, to test the relationship between institutional and pandemic influence. Institutional influence (II) is exogenous, and pandemic influence (PI) is an endogenous variable reliant on II. The model is constituted by 27 variables that consisted of 12 observed and 15 unobserved variables and is accompanied by 14 exogenous variables and 13 endogenous variables, as displayed by the SEM output. The SEM measurement model that executed CFA through SPSS AMOS is shown in Figure 2.

SEM output has showed that the model with a sample size of 922 is over-identified and recursive, with χ^2^ = 197.218 and *df* = 52 (>0), suggesting appropriateness for estimating various pathways (Khine, 2013). The sample size of 922 included in this study has justified enough sampling adequacy based on Hoelter's critical N displayed in the SEM output (Hoelter, 1983). By selecting the maximum likelihood estimation method (Byrne, 2013), SPSS AMOS automatically displayed estimations for all relationships with standardized and unstandardized estimates, which are presented in Tables 2 and 3.

Referring to Table 2, the R^2^ values for all endogenous variables ranged between 0.406 and 0.689, which indicated moderate (more than 0.50) to substantial (more than 0.75) strength in estimating endogenous variables (Cohen et al., 2013; Hair, 2009), except for proximity (C11) (*R*^2^ = 0.171, *β* = 0.413), which showed weak estimation strength but adequate estimates as data narrates to unpredictable human behavior (Chin, 1998). A higher value of standardized estimates (*β*) accumulated on institutional characteristics (C1 to C10) by virtue of institutional influence (II) proved to be a strong estimation. In the case of pandemic influence (PI) (*R*^2^ = 0.406, *β* = 0.637), the strength of determination is moderate with 40.6% of its variance explained on account of institutional influence (II). This means that if II increases by one standardized unit, PI will rise by 0.637 standardized units. Proximity (C11) is explained with 17 percent of its variance on account of PI. It will rise by 0.413 if PI goes up by one standardized unit (direct effect) and will rise by 0.263 standard units if II goes up by one standard unit (indirect effect). On the other hand, 77.3 percent of the variance in suitability under COVID-19 (C12) is estimated by PI. It will increase by 0.879 standardized units if PI goes up by one standardized unit (direct effect) and will increase by 0.560 if II goes up by one standard unit (indirect effect).

For the exogenous component, institutional influence (II) is assembled with 41.7% of its variance (CR = 12.039 > 1.96, *p* < 0.001), which is a moderate strength and reasonable value in behavioural research. Referring to Table 3, CR values associated with all pathways showing relationships between latent variable (II) and observed variables (C1 to C10) and between latent variable (PI) and observed variable (C11) are above 1.96. This further confirmed that strong convergent validity exists, as all scale items utilized in the CFA model have shown statistically significant loadings in hypothesized directions (Hair et al., 1998). In the case of relationships between two latent variables, II and PI, based on the *B* value, there is a positive relationship between them, indicating that if II goes up by one unit, then PI will go up by 0.942 units.

### 4.4 Model fitness and hypothesis validation

Fitness indices obtained for the measurement model of this study are noticed in accordance with various fitness indices recommended for SEM and hence support the plausibility of the relations among variables (Teo, 2014) (refer Table 4).

**Table 4:** Fitness of model.

Fitness indices	Recommendable limits for model	Measurement model under study	Literature support	Interpretation about Model fitness
χ^2^	Insignificant for N < 250	197.218 (Significant for N = 922)	(Anderson and Gerbing, 1988)	Good fit
Ratio χ^2^/*df*	<5 for (N > 500)	3.793 (N = 922)	(Marsh et al., 1988) (Marsh and Hocevar, 1985)	Good fit
Hoelter's critical N	N = 368 (minimum) for *p* < 0.001	N = 922	(Hoelter, 1983)	Good fit
TLI	>0.95	0.975	(Tucker and Lewis, 1973)	Good fit
CFI	>0.95	0.980	(Bentler, 1990)	Good fit
RMSEA	<0.5 (*p* of close fit >0.05)	0.55 (*p* of close fit >0.05) Fit of model is close	(Hu and Bentler, 1999)	Good fit

The research Hypothesis of this study that states there is no significant relationship between institutional influence and pandemic influence under the COVID-19 pandemic situation is tested by knowing the relationship PI←II (refer to Table 3 and Figure 2), which shows that this relationship is statistically significant in the positive direction (*B* = 0.942, CR = 16.434, *p* < 0.001). Hence, null hypothesis H_0_ is rejected, and alternative hypothesis H is accepted.

The SEM model has successfully presented a combination of a hypothetical path model and a CFA model which has statistically answered the research questions and validated the research Hypothesis of this study, henceforth, the research objective is achieved here. By comparing the indices required for good fit (refer to Table 4), the model – ‘choice influencing characteristics’ under the COVID-19 pandemic situation, as specified below has achieved a good fit.

χ2(52, N = 922) = 197.218, *p* < 0.001, CFI = 0.980, TLI = 0.975, RMSEA = 0.055 (CI90 0.047, 0.063, *p* = 0.146 > 0.05).

The model has thus demonstrated that the performance of the concept appears to be stable and robust, with all relationships that are hypothesized to be measuring what this study has set out to evaluate.

## 5 Statistical inference and discussions

This study has verified the influencing characteristics associated with EIs and the COVID-19 pandemic in regard to the selection of EIs during the COVID-19 pandemic situation. It has also verified the relationship between institutional influence and pandemic influence. Despite the fact that the performance of institutional influencing characteristics in pandemic situations is as usual as that in ordinary situations, it has incredibly affected pandemic influencing characteristics through proximity to the hometown and suitability under COVID-19. During the pandemic, institutional influence (β = 0.417) is significantly accumulated by the usual institutional influencing characteristics. This was also evidenced by several studies presented earlier in the pandemic.

The importance of location and locality (C1: μ = 3.992, *R*^2^ = 0.562, *β* = 0.750, *B* = 0.988) in making EI choice is evidenced by this study. During the COVID-19 pandemic, the ‘infected area’ related to coronavirus was the key anxiety for students; hence, they assessed it in terms of its spaciousness, airy ventilation, accessibility and suitability of facilities and amenities wherein it was situated. Similar findings were stated under nonpandemic conditions by Sovansophal (2019), who showed that a good location and locality are constructive in fetching enrolments.

Trust and beliefs are the key dimensions of image and reputation (Finch et al., 2013). During the pandemic, when almost nobody is aware of EI performance, students have no other options but to rely on them to provide suitable crisis management practices (Maringe and Gibbs, 2009) for continuing pedagogy that mitigate the risk of COVID-19. Furthermore, as the buying behaviour of customers in a pandemic crisis is believed to be a function of organizational reputation and trust (Coombs, 1998), EIs with a good image and reputation are more likely to be trusted under the COVID-19 situation. Because of this, students in this study have perceived image and reputation as an important characteristic (C2: μ = 4.120, *R*^2^ = 0.581, *β* = 0.762, *B* = 0.967) in selecting their EIs (Briggs, 2006; Wadhwa, 2016).

Faculty act as facilitators and mentors in preparing, interacting and motivating students to achieve their academic goals (Salami, 2007). Their support and motivation can be vital to improve students’ emotion and distress for better psychological well-being during the pandemic (Sood and Sharma, 2021). This is why the faculty profile (C3: μ = 3.964, *R*^2^ = 0.689, *β* = 0.830, *B* = 1.058), as usual, is treated as an important influencing characteristic that facilitated the choice of EIs. Bao (2020) documented similar importance in terms of the importance of faculty assistance in impacting and sustaining higher education during the COVID-19 pandemic period.

Alumni profile is another causative characteristic of EIs, vital for prospective students and their family in making EI choice. Alumni's overall status, such as their reputation gained after graduation (Ho and Hung, 2008) and their employment position (Kalimullin and Dobrotvorskaya, 2016), holds significance during pandemic situations. Students can analyse the risk involved in studying selected EI with these kind of benefits they will receice after graduating from that EI. Importance of alumni profile (C4: μ = 3.906, *R*^2^ = 0.667, *β* = 0.817, *B* = 1.062) appeared to be in accordance with the results of Ho and Hung (2008) in making EI choice.

As the majority of entry-level jobs in the engineering profession during the pandemic are diminishing, campus placements can only provide students with a breakthrough that can make their engineering career worthwhile. During the pandemic, campus placement activities of EIs can offer rewarding benefits in terms of skill development that make students competitive in the world and can offer better employment opportunities in the job crisis happening during the pandemic. This is what students under this study might have perceived and hence campus placement (C5: μ = 3.979, *R*^2^ = 0.623, *β* = 0.789, *B* = 1.003) of EIs is proven to be a governing characteristic in deciding EI choice (Malgwi et al., 2005; Matusovich et al., 2020a).

This study has revealed that quality education (C6: μ = 3.937, *R*^2^ = 0.671, *β* = 0.819, *B* = 1.075) is an important institutional characteristic in deciding EI choice. The notion of ‘quality’ in higher education is a function of tangible facilities, intangible services and human relations. Students under this study acknowledged its importance in delivering an excellent learning atmosphere during the COVID-19 pandemic situation. The need for such an atmosphere was also noted by (Zuhairi et al., 2020).

Infrastructure and facilities (C7: μ = 3.911, *R*^2^ = 0.680, *β* = 0.824, *B* = 1.063) is a fundamental support of the higher education system that is rendered through its suitability, accessibility and affordability to continue pedagogy during pandemic situations (Raaper and Brown, 2020). Hence, the students under this study are influenced in making their EI choice which is supportive to the findings of Sahu et al. (2013).

During pandemic situation, pedagogy must be delivered by following preventive measures and mandatory standards (Cheng et al., 2020) which requires strong measures on safety and security for students’ overall wellbeing. Today, safe and secured arrangements are contemplated as personal protection shields for students during pandemic situations. For this reason, this study has observed safety and security (C8: μ = 3.972, *R*^2^ = 0.660, *β* = 0.812, *B* = 1.019) as a key influencing characteristic in making EI choices which is mentioned by Calitz et al. (2020).

Curriculum delivery during pandemic is the most difficult challenge for engineering studies, and redesigning it via online, onsite or hybrid modes in pandemic situations is an urgent need (Cahapay, 2020) that reduces the burden of cost, workload and eases mental stress, however keeps the momentum going. Therefore, curriculum delivery (C9: μ = 3.929, *R*^2^ = 0.667, *β* = 0.816, *B* = 1.037), as evinced by Moogan and Baron (2003), is a key influencing characteristic of EIs in making choice decisions.

The importance of value for money as stated in previous studies (Ivy, 2008; Joseph et al., 2005; Kotler and Fox, 1985) is sustained by this study. Cost-effectiveness, convenience, time, and efforts spent are more vital, as they relate directly to the mental and health conditions of students. For this cause, value for money (C10: μ = 3.764, *R*^2^ = 0.494, *β* = 0.703) has a positive influence in directing students’ decision making.

Referring to proximity to hometown (C11: μ = 3.329, *R*^2^ = 0.171, *β* = 0.413, 0.263, *B* = 0.562), this study has indicated that it has affected students' choice. Proximity is controlled by pandemic as well as institutional influence in a positive direction. This means that if pandemic influence increases, importance of proximity also increases. This further justified that EIs situated near students’ markets are in a better position to be selected by local students (Matusovich et al., 2020b), as decrease in the distance travelled saves time and cost for the family, and sustains health-related safety and security during the pandemic. In other way, this supports the findings of Mok et al. (2021), who realized that institutions that are placed at a far distance have more to work-on in terms of reframing policies to attract students during the pandemic.

The parameter, influence of suitability under the COVID-19 pandemic, is employed and analyzed for the first time through this study. It denotes an environment that brings normality into engineering pedagogy with the ease of accessibility and suitability during the COVID-19 situation by following social distancing standards. It (C12: μ = 3.502, *R*^2^ = 0.773, *β* = 0.879, 0.560) is proved to be a major contributing factor for pandemic influence and is also affected by institutional influence.

Overall, pandemic influence is well administered under the impact of institutional influence. It is thus confirmed that traditional choice influencing characteristics strongly direct students' perceptions about suitability of EIs under COVID-19 pandemic situations. It can be summarized that, EIs with better repositioning in terms of its’ choice influencing characteristics will be perceived to be greater suitable under pandemic situation.

## 6 Implications, suggestions, and contribution

According to the findings of this study, traditional institutional influencing characteristics along with the consideration of pandemic situation must be reconsidered to enhance suitability under pandemic conditions. During the pandemic, institutional characteristics seem to have strong and positive impressions on pandemic influence which includes suitability under COVID-19 and proximity to hometown. Thus, this study has explored how existing institutional characteristics can control situational influence. The following managerial implications and suggestions are envisioned for the effective performance of EIs during the pandemic by reframing institutional characteristics.

During the pandemic, institutional governance and students centric services that keep the interest of students ongoing, minimize their academic loss, create a feeling of being affiliated and justify them as ethical engineers are very important aspects in developing a high prestige and high reputation of EIs (Gill et al., 2018). Furthermore, providing quality infrastructure and facilities along with effective crisis management measures (Maringe and Gibbs, 2009) during the pandemic will trigger positive insights into the quality of EIs (Hemsley-Brown and Oplatka, 2015a).

With one action, EI can witness two-fold benefits during the pandemic. First, providing quality education and services will positively improve image and reputation (Khoi et al., 2019). Second, it will build trust in EIs' commitments to provide quality services. It will also achieve students' reliability and confidence in quality provisions rendered by EIs during the pandemic. EIs further need to create co-creating mechanism for providing and processing vital information about their offers for informed choice decisions (Mogaji and Maringe, 2020). EI stakeholders, such as faculty, existing and alumni students, are the direct sources of spreading ‘word-of-mouth’ about ‘suitability’ of EIs during the pandemic.

Due to the immobility of physical assets, EIs have little to work on proximity once established. However, as this study has predicted the importance of proximity to the hometown, it becomes binding on local institutions to provide excellent educational services with social distancing norms to grab new enrollments. The success of EIs will be dependent on how far it creates a ‘house of reliance’ (Nandy et al., 2021) for them. All such efforts will ultimately develop institutional image (Manzoor et al., 2020) and long-lasting relationships (Clark et al., 2017), which is essential in creating future markets for EIs during pandemic situations.

Nevertheless, EIs should initiate their repositioning by following pandemic guidelines issued by the government and apex authorities from time to time. If the pandemic carries with us for a long life, then the institute will have to open up other options, such as small campuses and relocation in remote places (Gross, 2020).

To the best of our knowledge, this study is the first to present insights regarding the performance of choice influencing characteristics that are responsible for selecting EIs during the pandemic situation. It perhaps is the first study to come up with new look-out ‘pandemic influence’, which is noticed to have a significant utility in evaluating choice characteristics under pandemic conditions. It has successfully examined and explored the relationship of suitability and proximity with the pandemic influence as well as traditional institutional influence. Next, it has come with significant evidence that traditional institutional influencing characteristics are positively related to pandemic influence. This is the main contribution of this study.

The study has provided substantial hopes for policy makers. As it has firmly established and deeply rooted in most challenging task of administering new enrollments. EIs will have to reposition themselves to normalize pandemic influence by tuning institutional characteristics. The SEM model of this study can be a measurement tool for EIs to stay ahead in competitive markets. Accordingly, the study has added new and substantial materials and thus has made several key contributions to the existing body of knowledge.

## 7 Conclusion

Choice influencing characteristics associated with EIs should be tailored to covert ‘willingness’ of aspirants into their ‘acceptance’. Although attracting new admissions to EI campuses before the pandemic was a difficult task, the current study analytically mapped the influence of institutional characteristics that regulate pandemic influence under pandemic conditions. The study of 922 newly admitted students in thirty nine engineering institutions situated in North Maharashtra Region of India, has revealed that traditional institutional characteristics governing choice decisions have a predominant effect on pandemic influence. The findings have also confirmed that the proximity to hometown (17.1% of variance) and suitability of EIs under pandemic conditions (77.3% of variance) are the key characteristics that have statistically contributed in governing pandemic influence. Specifically, the study has exposed a statistical relationship between institutional influence and pandemic influence. The variance of 40.6% in pandemic influence is explained by institutional influence with strong predicting positive estimates (*B* = 0.942).

To culminate at this moment, it is dubious that how EIs will be weathering a ‘new normality’ during the pandemic. The answer to this question is very reliant on EI's resilience in reframing student-centric practices that govern suitability under pandemic conditions for prospective enrollments. For the moment, it is time to make a "change for the better" that intensifies demand for engineering education and expedites choice making decisions during the pandemic situation. The findings of this study may bring ‘normality’ to ‘new’ enrollments and can become a revolutionary transformation in the future ahead.

## 8 Limitations and future research

Like any research that employs a limited sample, this study is restricted to the fact that it deals with a single context, the North Maharashtra region of India, so that its findings cannot be directly generalized. All things considered, the current study's sincerity and relevance lies in exploring the relationship of ‘pandemic influence’ with traditional influencing characteristics. Realizing these facts, plenty of research doors are opened for investigating institutional influence in regard to other disciplines in higher education in different regions. Such future studies may report various relationships, as choice characteristics and pandemic impact vary with the region wherein the institutions are situated; consequently, various perspectives on pandemic influence and suitability of the institutions can be acquired under pandemic conditions.

Next, the survey was conducted during the COVID-19 pandemic, and the findings may not be similar to a typical situation. Another fact is that the choice process for students mainly begins during their precollege days. In India, as this pandemic has arrived in 2020, some students may not have much exposure to its influence. Henceforth, future research is encouraged periodically but frequently that includes a choice process over the entire pandemic period. Pandemic influence and suitability under COVID-19 are utilized for the first time in this study to give general ideas about their relationships. Although sufficient progress on choice characteristics has been detailed in the first attempt, a more refined and detailed scale can be developed in future research.

## Declarations

### Author contribution statement

Prashant Mahajan and Vaishali Patil: Conceived and designed the experiments; Performed the experiments; Analyzed and interpreted the data; Contributed reagents, materials, analysis tools or data; Wrote the paper.

### Funding statement

This research did not receive any specific grant from funding agencies in the public, commercial, or not-for-profit sectors.

### Data availability statement

Data included in article/supplementary material/referenced in article.

### Declaration of interests statement

The authors declare no conflict of interest.

### Additional information

No additional information is available for this paper.
